# Kinetics of Bulge Bases in Small RNAs and the Effect of Pressure on It

**DOI:** 10.1371/journal.pone.0042052

**Published:** 2012-08-20

**Authors:** Pradeep Kumar, Jean Lehmann, Albert Libchaber

**Affiliations:** Center for Studies in Physics and Biology, Rockefeller University, New York, New York, United States of America; Universite de Sherbrooke, Canada

## Abstract

Due to their self-catalytic properties, small RNAs with bulge bases are hypothesized to be primordial molecules which could form elementary translation systems. Using molecular dynamics simulations, we study the binding propensity of small RNAs by calculating the free energy barrier corresponding to the looped out conformations of bulge bases, which presumably act as the binding sites for ligands in these small RNAs. We find that base flipping kinetics can proceed at atmospheric pressure but with a very small propensity. Furthermore, the free energy barrier associated with base flipping depends on the stacking with neighboring bases. Next, we studied the base flipping kinetics with pressure. We find that the free energy associated with base looping out increases monotonically as the pressure is increased. Furthermore, we calculate the mean first-passage time of conformational looping out of the bulge base using the diffusion of reaction coordinate associated with the base flipping on the underlying free energy surface. We find that the mean first-passage time associated with bulge looping out increases slowly upon increasing pressures 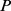 up to 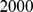 atm but changes dramatically for 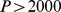 atm. Finally, we discuss our results in the light of the role of hydration shell of water around RNA. Our results are relevant for the RNA world hypothesis.

## Introduction

RNA molecules are very diverse both structurally and functionally [1], [2]. Apart from having the regular helical purine-pyrimidine base pairs, RNA molecules are also found to have many other secondary structures (motifs) such as loops, knots and bulges etc [3], [4]. The presence of such structural motifs is found to play a role in binding of different molecules to RNA [5]. For many protein binding RNAs, it was found that the frequency of adenosine bulge at the binding site is very high. The presence of a bulge may change the conformational flexibility of an RNA [6], [7] and hence more internal surface area of RNA is available for any chemistry. Moreover, the presence of the bulges does not only change the conformational flexibility but the bulges themselves may just flip out exposing the internal regions of a RNA to solvent and ligands. It has been shown that the bulge base looping out is highly sensitive to the bulge bases and their neighbors [6], [7], which makes the question of generality of any picture of base bulge looping out difficult.

RNA is hypothesized as a primordial molecule due to its ability to form efficient catalysts and its similarity to DNA, where it can act as a molecular information machinery [8]. Yarus and coworkers have shown that RNAs, as small as 29 nucleotides, can undergo self-aminoacylation with aminoacyl adenylate (aa-AMP) as a substrate [9]. Lehmann and coworkers further studied the effect of base composition and length of the 3′ extension on the aminoacylation rate of these small RNAs [10]. These studies indicate that these small RNAs may act like primitive tRNA. Furthermore, due to rich chemistry of the reactive gases, and dissolved elements, a reminiscence of the early earth, hydrothermal vents are hypothesized to offer conditions viable for the origin of life [11], [12]. Thermal vents naturally provide thermal gradients over small to very large length scales. Besides temperature gradient, the pressure near vents is much higher compared to atmospheric pressure. Hence, if a small primitive tRNA is selected at these thermodynamic conditions then they should also be able to carry out self-aminoacylation at high pressures.

The active site in the small tRNA-like molecules, that is believed to bind to aa-AMP, consists of AG/AG bulge in the stem [9], [10]. The binding of aa-AMP proceeds by flipping of the bulge bases providing space for adenosine of the aa-AMP to stack in. Recent state of the art computer simulations of small RNAs have shed light on the bulge base looping out processes [13]–[15] in few of the possible cases. Specifically, these works have studied the free energy barriers associated with bulge base looping out process [13]. For example, A. Barthel and M. Zacharias studied the kinetics of bulge base looping out of a single uridine and adenosine bulge structures. Specifically, they calculated the free energy barrier corresponding to torsional deviation which measures the degree of looping out of bulge bases from the local helical plane. They find that the conformational free energy change in the case of adenosine bulge in a complete looping out process is higher by 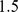 kcal.mol^−1^ as compared to the uracil bulge [13], suggesting that in a base nonspecific binding process, a structure with single uracil bulge base would have higher propensity to flip out of helical plane. Although a wealth of literature is available on the base looping from the helical plane at ambient conditions, the changes in the kinetics of bulge base flipping is unexplored at conditions away from ambient conditions.

Related is the question of the effect of RNA hydration on RNA kinetics. For binding to proceed, the bulge base has to flip out of the local helical plane, and so has to overcome both the bending rigidity and solvation energy barrier to solvate in water. The solvation of different substances in water is a widely studied problem [16]–[21]. Indeed, studies of apolar solutes in water shows an elliptic region in the pressure-temperature plane in which water behaves as a bad solvent and hence less solubility of these substances [22]. Moreover, It is known that water's hydrogen bond network and so the local structure of liquid water changes upon changing thermodynamic conditions, giving rise to anomalous changes in the dynamics and thermodynamics of water and aqueous systems. The structural stability and kinetics of proteins (where structural stability usually implies kinetically functional) is a function of pressure and temperature. The solvation barrier of apolar solutes in the case of proteins plays an important role both in the hydrophobic collapse of the polypeptides [17], [22] as well as the stability of these structures as a function of pressure and temperature. The effect of the RNA hydration on the kinetics of RNA has not been given much attention.

In this paper, we first compare the propensity of bulge base looping out of a double strand RNA (dsRNA) for three different sequences: (i) with a single adenosine bulge in sequence 5′GGGGAGG3′-5′CCCCCC3′, and (ii) with a single adenosine bulge in sequence 5′CCCCACC3′-5′GGGGGG3′, and (iii) an AA-bulge in 5′GGGGAGG3′-5′CCACCCC3′. Next,we study the effect of pressure on the bulge base looping out for sequences (i) and (ii) with a single A-bulge. The organization of the paper is as follows. In section “Methods”, we discuss the computational method, in section “Results”, we present the results for the free energy barrier associated with torsional deviation of the adenosine bulge base from the local helical backbone at both atmospheric and elevated pressures. Then, we next present a mean first-passage time calculation associated with bulge base looping out process, and the effect of hydration on the kinetics. Finally we summarize and discuss our results in the “Discussion” section.

## Methods

**Figure 1:**
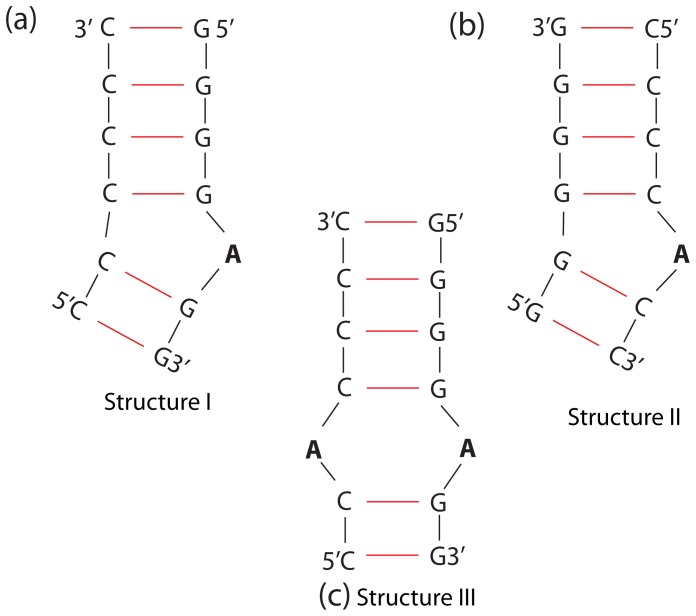
2-dimensional schematics of the double strand RNA structures used in this study. (a) with a single A-bulge in 5′GGGGAGG3′-5′CCCCCC3′ (structure I), (b) with a single A-bulge in 5′CCCCACC3′-5′GGGGGG3′ (structure II), and (c) with an AA-bulge in 5′GGGGAGG3′-5′CCCCCC3′ (structure III).

The energy minimized starting structures of three different RNAs (i) with a single A-bulge embedded between (GC)_2_ (5′-GGGGAGG-3′/5′-CCCCCC-3′) (Fig. 1(a)), (ii) with a single A-bulge embedded between (CG)_2_ (Fig. 1(b)), and (iii) with an AA-bulge (5′GGGGAGG3′-5′CCCCACC3′) embedded between (GC)_2_ (Fig. 1(c)) were created using the NAB/Nucgen module of the Amber10 program suite. The RNA structures were then solvated in 4000 TIP3P water molecules such that there was about 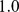 nm space left between the boundary of the box and the RNA molecule. For electro-neutrality, appropriate number of Na+ counter ions were added to the system. Energy minimizations were carried using the steepest descent (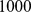 steps) in GROMACS3.3.3 [23], [24] with keeping the RNA atoms fixed. GROMACS is an all purpose molecular dynamics program which has been used to study various molecular systems [23]. The choice of GROMACS for our studies were mostly the convenince of it for doing dihedral restraint simultions. Any other programs that can provide a nucleic forcefield implementation, such as Amber [25] or NAMD [26] could have been used. Simulations were carried out using the GROMACS3.3.3 program with Amber99 force field. Equations of motion were integrated using a time step of 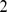 fs with periodic boundary conditions. The long range electrostatic interactions were treated with the particle-mesh-ewald (PME) method. After minimization, the system was slowly heated to temperature T = 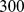 K with positional restraints.

After the position restrained simulations, unrestrained Molecular Dynamics (MD) was carried out at four different values of the pressures 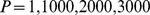 atm. Thermal equilibrium at a constant temperature 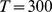 K and different pressures was achieved using Berendsen thermostat and barostat respectively. The final equilibrated conformation was then used for as the starting conformation for umbrella sampling at different pressures.

**Figure 2:**
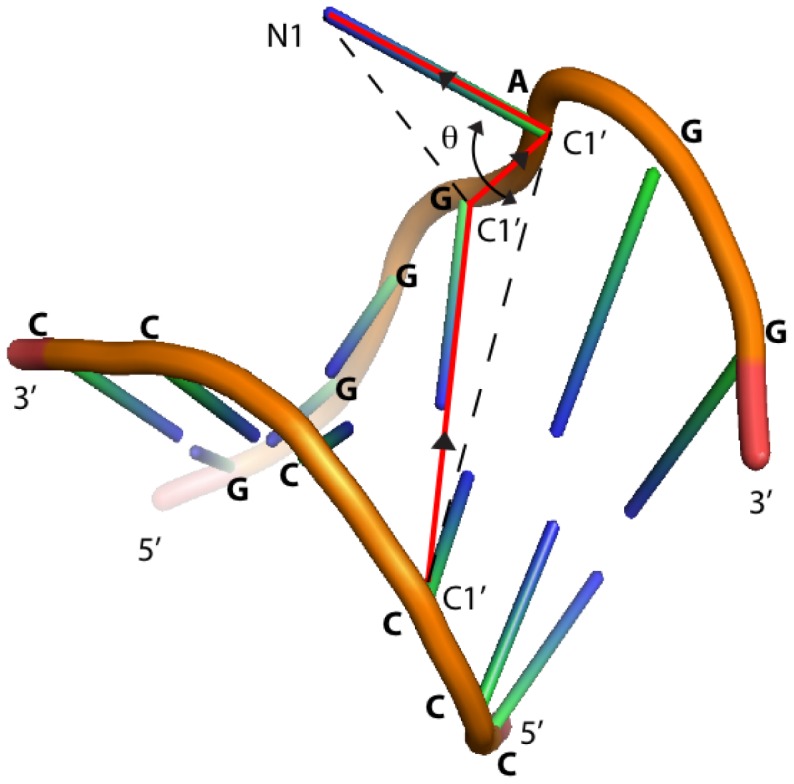
Stick representation of the three-dimensional structure of 5′GGGGAGG3′-5′CCCCCC3′ RNA and the definition of the torsional angle (C1′C1′C1′N1) chosen as the reaction coordinate for the calculation of free energy of bulge base looping out from the local helical plane. The torsional angle 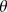 is represented by red lines with arrows, and is the angle between the planes formed by C1′C1′C1′ and C1′C1′N1.

To quantify the relative propensity of binding of RNA at different pressures, we chose the dihedral angle 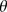 (C1′C1′C1′N1) (see Fig. 2) as the reaction coordinate for calculation of free energy. Since the conformational changes are very slow and an equilibrium sampling of torsional angles require much larger time scales than computationally feasible, we use biasing potential to calculate the free energy. Umbrella sampling method was used to calculate the relative free energy of bulge base looping out conformations of the RNA shown in Fig. 2. Harmonic umbrella biasing potentials 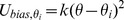 with a force constant 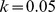 kcal.mol^−1^.deg^−2^ were distributed uniformly along the reaction coordinate 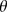 at an interval 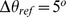. Consecutive sampling windows of 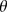 were started from equilibrium structure of last run. For each values of the umbrella sampling window, we run a 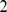 ns simulation and record the value of 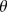 every 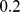 ps. The final potential of mean force (PMF) was calculated using the WHAM (weighted histogram method) [27]. The unbiased probability distribution 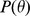 at a given temperature 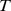 under WHAM is given by
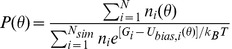
 where 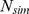 is the number of sampling window (simulations), 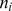 is the number of counts in the bin associated with 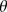, 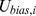 is the biasing potential, and 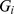 free energy from simulation 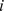 and is given by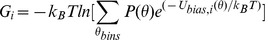 where 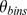 is the number of bins for the individual sampling window. The equations 1 and 2 are iterated to obtain the self consistent value of 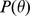. The value of 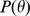 depends on the time scale of simulations and hence long simulations are needed for a good convergence of the free energy.

## Results

### Kinetics of bulge base at ambient and elevated pressures

**Figure 3:**
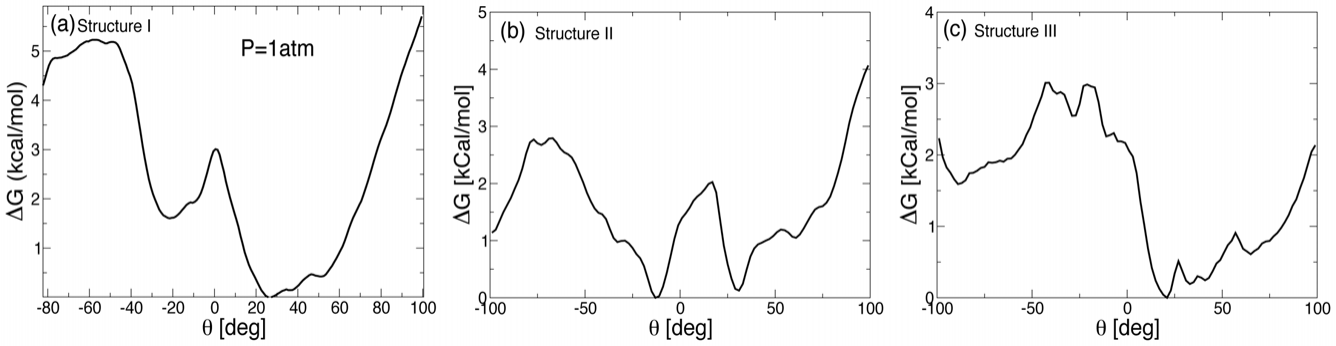
Free energy as a function of the torsional angle 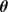 at 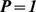 atm, and 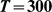 K. (a) 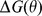 for single A-bulge at 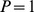 atm in structure I. The free energy profile associated with bulge flipping has two minima as found in earlier studies [13], separated by an energy barrier of 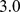 kcal.mol^−1^. (b) 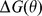 for a single A-bulge in structure II. 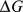 corresponding to base looped out state (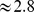 kcal.mol^−1^) is the minimum among the three case studied here. (c) 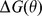 for AA-bulge in structure III. Absence of two minima suggests that in this case there is no formation of base triplet configuration. The free energy barrier associated with complete base looped out state is lower compared to single A-bulge case in (a).

In Fig. 3(a), we show 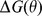 as a function of 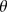 for the RNA structure I with a single 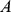-bulge at 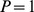 atm. Negative value of 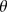 corresponds to deviation towards minor groove while the positive values correspond to deviation towards major groove. We find that 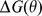 has characteristic two minima centered around 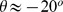 and 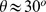 as reported in earlier studies of single A bulge [13]. Note that the definition of the torsional angle 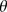 is different from the one used Ref. [13] and hence different values of 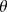. As we can see from Fig. 3 (a), the orientation of the A-bulge at more stable minimum is tilted slightly along the major groove while the second minimum at 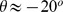 is presumably due to the base triplet formation with the neighboring bases. The free energy difference between these two minima is 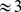 kcal.mol^−1^, suggesting that although 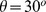 is relatively a more stable minimum configuration, the thermal fluctuations at 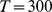 K (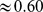 kcal.mol^−1^) is sufficient enough for the bulge to get displaced of the free energy minimum configuration. Due to a large free energy barrier (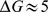 kcal.mol^−1^) a complete looped out conformation of single A-bulge is less favorable and hence consistent with experimental fact that RNA with single A-bulge does not show appreciable aminoacylation (unpublished work). In Fig. 3 (b), we show 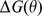 for a single A-bulge looping for structure II. In this case, the free energy barrier associated with complete looped state is smaller compared to structure I. Moreover, 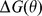 exhibits two minima, one corresponding to the energy minimum stacked configuration and the other corresponding to the base-triplet formation at about 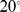 along the minor groove. In Fig. 3(c), we show 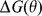 as a function of 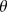 for the RNA structure III with an AA-bulge at 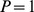
atm, and 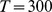 K. Compared to single A-bulge in structure I (Fig. 3), we find that in the case of AA-bulge, 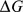 for a complete flipped out configuration is smaller (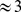 kcal.mol^−1^). Moreover, the secondary minimum as seen in the the case of single A-bulge (structure I, and II) is absent. Hence, the rate of base flipping would be enhanced for AA-bulge compared to single A-bulge base in structure I.

**Figure 4:**
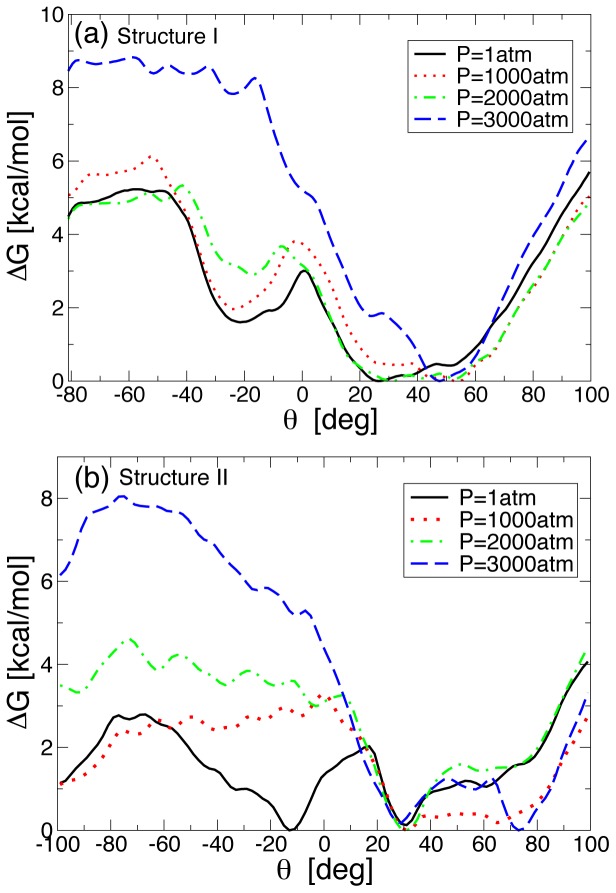
Free energy as a function of the torsional angle 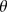 for different pressures. (a) Free energy 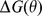 for structure I at 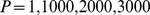 atm, and 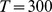 K. (b) Analogue of Fig. 4(a) for structure II. The free energy barrier between the two minima slowly disappears upon increasing pressure, suggesting that the transient barrier that is produced at atmospheric pressures due to the partial base triplet hydrogen bonding of the bulge base with the neighboring bases is broken at higher pressures. Moreover, in both the cases 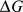 corresponding to complete flipped out state shows a sharp change for 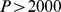 atm.

We next studied the effect of pressure on the kinetics of bulge bases in structure I and structure II. Fig. 4(a) shows 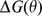 for A-bulge in structure I as a function of 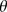 for different 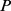 at 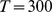 K. We find that for pressures up to 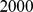 atm, 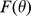 has the characteristic two minima as we find in the case of 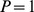 atm. However, as the pressure in increased, the minimum at 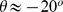 becomes shallower and disappears for 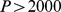 atm, suggesting that at 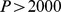 atm the base triplet formation of the bulge base with the neighboring bases does not occur during the looping process. Moreover, the free energy barrier between the two minima changes just a little upon increasing pressure for 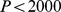 atm. For 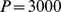 atm, free energy barrier for the flipped out state changes drastically where 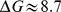 kcal.mol^−1^, suggesting that the propensity of single A-bulge base looping out from the local helical plane would decrease upon increasing pressure and so the binding propensity of incoming ligands.

Fig. 4(b) shows 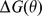 for A-bulge in structure II as a function of 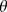 for different 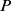 at 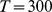 K. As the pressure is increased, the base triplet minimum seen at 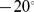 disappears. The free energy barrier associated with a complete flipped out state of the bulge base progressively becomes larger with pressure exhibits sharp jump at 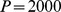 atm. At the largest pressure, 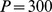 atm, studied here, 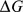 corresponding to complete flipped out state is 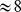 kcal.mol^−1^. Comparing Fig. 4 (a) and (b), we find that the effect of pressure up to about 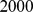 atm is rather moderate on the kinetics although the rate of bulge base flipping decreases monotonically with pressure. Moreover, in both cases, whether the bulge base A is embedded with neighboring Guanine bases or Cytosine bases, the qualitative effect of the pressure on the kinetics remains the same.

**Figure 5:**
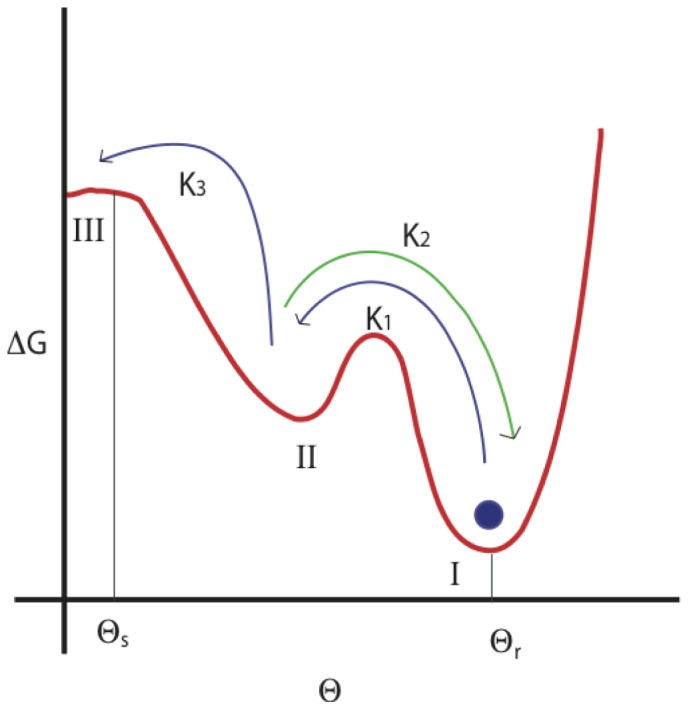
Schematic of free energy as a function of the torsional angle 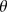 used for the calculation of the effective time scale of bulge base flipping kinetics.

In order to calculate the effective rate 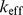 of transition from a stacked to looped out conformation, we use Langevin equation [28], [29]. Assuming the diffusion of the reaction coordinate 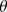 on an underlying free energy surface the dynamics of 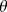 is governed by
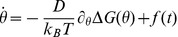
 where 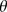 is the torsional deviation and 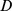 is the diffusion constant, 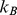 is the Boltzmann constant, and 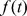 is the thermal noise with zero mean, 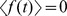 and delta function correlation, 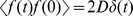. In the high friction limit, the probability 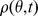 of finding the system with reaction coordinate 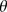 after time 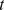 is given by the Smoluchowsky equation: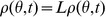 where 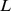 is the Fokker-Planck operator given by 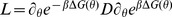 and 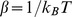. The mean first-passage time 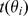 associated with crossing the barrier from any coordinate 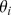 to final state 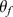 is given by (see Fig. 5)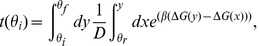 where 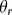 and 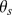 denote the reflecting and absorbing boundaries respectively. We choose 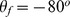 as the final looped out state and initial state is chosen to the values of 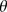 where the free energy curve has the deepest minimum for respective pressures. The effective rate 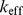 of transition from 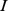 to 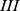 would then be given by 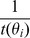. The reflecting boundary was chosen to be at 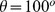 where the relative free energy is 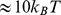. Using Eq. 5, we calculate the value of 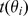 for different pressures. We list the values of 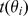 in table 1, where 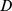 is measured in 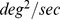. We find that 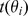 increases upon increasing pressure within the error bars and increases sharply for 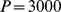 atm.

**Table 1:** Mean first-passage time of base flipping at different pressures.

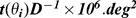		
Pressure	Structure I	Structure II
1 atm	2.16	0.023
1000 atm	9.81	0.075
2000 atm	5.38	0.367
3000 atm	1018	324

### Hydration shell and base flipping of RNA

**Figure 6:**
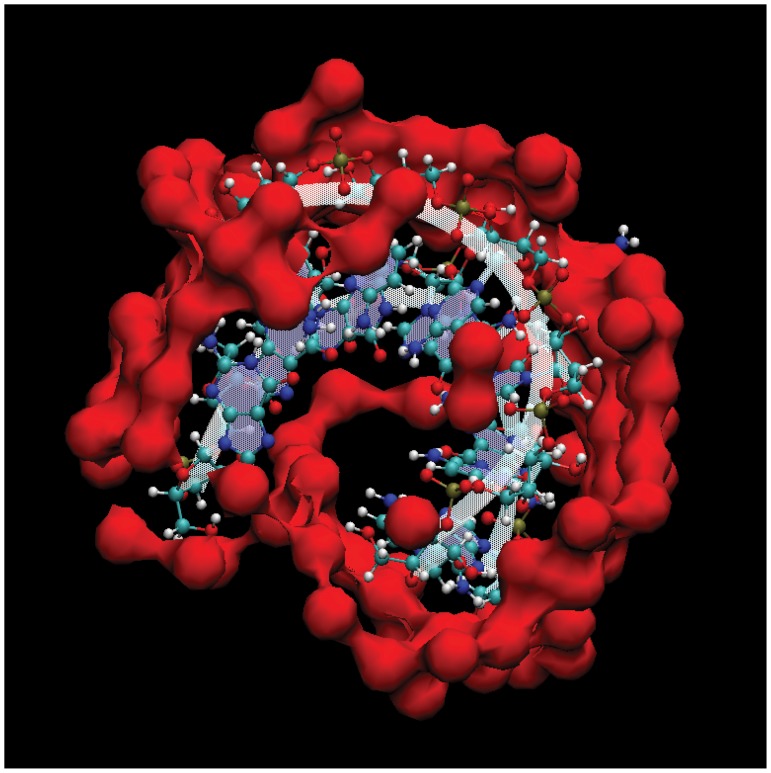
A typical hydration sheath around RNA. Water molecules are represented by a surface plots and the hydration shell is obtained as mentioned in section IV.

As we have seen in the sections above, the base flipping kinetics changes as the pressure is increased – namely, the free energy barrier for the bulge base to flip out increases with pressure. Moreover, the transient barrier which presumably is due to the base triplet formation of the bulge base with the neighboring bases disappears at pressures 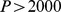 atm. We note that 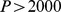 atm is the pressure where most of the anomalies of liquid water disappears and also the pressure at which hydrophobic barriers for small molecules in water tend to vanish. Motivated by this we looked at the structure of the solvation shell (first hydration shell) of water around RNA for different pressures. We show a typical hydration shell around RNA in Fig. 6. The hydration shell is calculated by finding all the water molecules within a distance 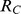 of RNA molecule. We choose 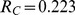 nm as the first minimum in the radial distribution function of RNA and oxygen of water molecules (not shown here). We find that 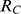 is independent of the pressure.

**Figure 7:**
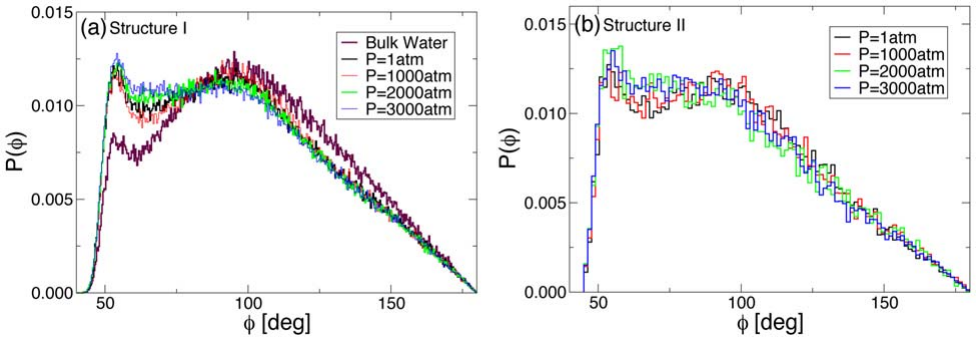
Probability distribution function 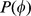
 **of the OOO-angle** 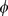 **in the first hydration shell at various pressures for (a) for structure I, and (b) for structure II.** Note that a comparison with 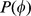 for bulk water at 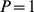 atm suggests that hydration shell is more disordered and this disorder increases monotonically upon increasing pressure.

We first looked at whether the observed change in the pressure dependence of the kinetics is a result of ordering of water around RNA. To this effect, we calculated the OOO-bond angle 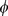 and its distribution 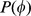 of a central water molecule and its nearest neighbors in the hydration shell. For ordered liquid water 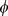 is very close to the tetrahedral angle 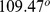. In Figs. 7 (a) and 7(b), we show 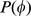 for structures I, and II for pressures 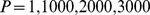 atm, and 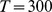 K. For a comparison we also plot 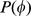 for bulk TIP3P water at 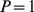 atm and 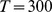 K. We find that, the second peak corresponding to more ordered water of the distribution 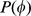 shifts to smaller values of 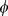, suggesting that the water monotonically disorders upon increasing pressure. Moreover, we do not find any significant sharp changes in 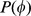 which could be associated with sharp change in the free energy barrier observed at 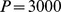 atm.

To quantify the ordering/disordering of water molecules around 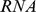, we use the tetrahedral order parameter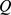 [30]–[32]. Tetrahedral order parameter 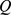 quantifies how close a given water molecule and its first shell neighbors form a structure close to a tetrahedron. In general, 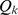 of 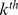 molecules is defined as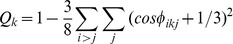 where, the indices i, and j run over all four neighboring molecules and 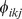 the OOO-angle formed between the oxygens of central molecule k and neighbors i and j.

Since, we only consider a thin hydration shell, the expression for ensemble average 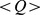 can be written as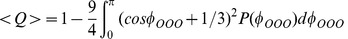 In table 2, we list the average tetrahedral order parameter 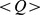 of the hydration shell for different pressures. For a comparison, we also compute 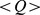 for bulk water. Table 2 lists values of 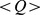 for different pressures. We find that, 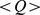 monotonically decreases upon increasing pressure and no sudden change in 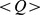 is seen at 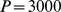 atm.

**Table 2:** Average tetrahedral order parameter 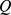 of the first hydration shell of water around RNA at different pressures.

Average Tetrahedral Order Parameter 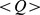			
Pressure	Bulk Water	Structure I	Structure II
1 atm	0.46	0.396	0.37
1000atm		0.37	0.36
2000 atm		0.36	0.32
3000atm		0.38	0.34

**Figure 8:**
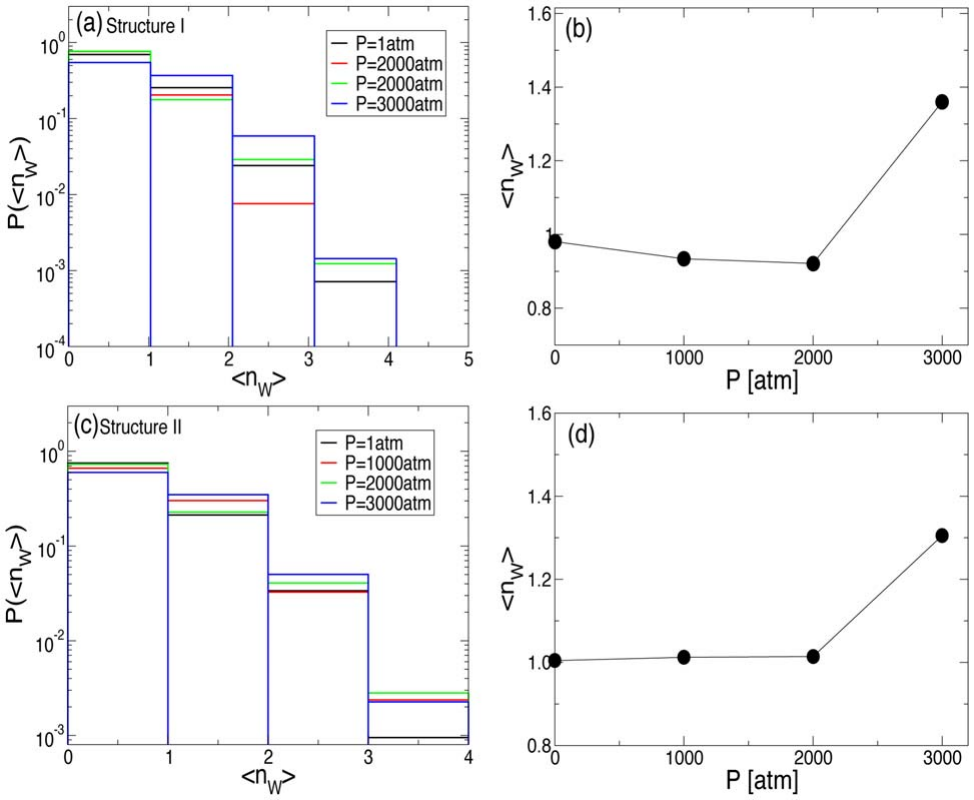
Probability distribution and average number of water molecules in the first hydration shell of the bulge base. (a) Probability distribution 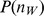 of water molecules in the first hydration shell around the A-bulge base in structure I for different pressures at 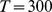 K for structure I. Note that the probability of 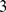 or 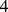 water molecules increases as the pressure is increased. (b) Ensemble averaged 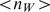 as a function of pressure. (c) Analogue of Fig. 8(a) for structure II. (d) Analogue of Fig. 8(b) for structure II.

Since we did not see any sudden change in the ordering of hydration shell around RNA that might lead to the base flipping barrier observed at 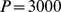 atm, we next studied the hydration shell of the bulge. In Figs. 8(a) and (c), we show distribution 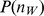 of number of water molecules 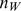 in the hydration shell of the bulge for structures I, and II respectively. Surprisingly, we find that at high pressures, the average number of water molecules in the first hydration shell of the bulge base increases, from an average of about 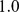 to 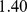 (see Fig. 8(b) and Fig. 8(d)). Moreover, we find that the distribution of water molecules around the bulge base shows significant probability of finding 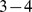 water molecules. To this end, we suggest that the increased barrier of base flipping and the disappearance of the transient barrier in the free energy barrier is due to the presence of increased water molecules in the solvation shell around the bulge base. At high pressure, presence of more water molecules in the hydration shell of the bulge, which might have penetrated from the major groove, may have led to stabilizing the stacking of the bulge base. Increased free energy barrier at high pressures could be due to energetic factors such as hydrogen bonding with the increased water molecules in the hydration shell, or entropic factors. In general it is a contribution of both factors. In future, relative roles of enthalpic and entropic barriers must be investigated to shed more light on the sharp change in the kinetics observed at high pressure.

## Discussion

In summary, in this paper we have investigated the effect of neighbor stacking and pressure on the kinetics of an Adenosine bulge base embedded between (GC)_2_ in 5′GGGGAGG3′-5′CCCCCC3′ (structure I), and embedded between (CG)_2_ in 5′CCCCACC3′-5′GGGGGG3′ (structure II), and AA-bulge embedded between (GC)_2_ in 5′GGGGAGG3′-5′CCACCCC3′ (structure III). Specifically, we calculate the free energy barrier associated with the base looping out from the local helical plane of the RNAs. We find that a single A-bulge base embedded between (GC)_2_ has a much larger free energy barrier of complete looping out compared to both AA-bulge embedded between (GC)_2_, and A-bulge embedded between (CG)_2_. Among the three structures studied here, the free energy of looping out of A-bulge is minimum for A-bulge base embedded between (CG)_2_. Our results indicate the importance of stacking interactions with neighboring bases in determining the rate of base flipping. It further suggests that certain structural features of the bulge bases may facilitate the binding of small molecules to small RNAs. Usually, an A-bulge base with weaker neighbor stacking will have faster rate of flipping as compared to AA-bulge with the same neighbor stacking. We have studied only a few simple cases here. A complete underlying physical picture could only come from looking at various structural motifs and identifying the structural features that may increase or decrease the binding propensity of small RNAs.

Motivated by the fact that near the thermal vents the pressure is very high, we next studied the effect of pressure on the kinetics of A-bulge base for two configurations. We find that, upon increasing the pressure, the propensity or likelihood of base flipping decreases. At pressure 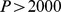 atm, we see a sharp increase in the free energy barrier. Further, we calculate the time scale of flipping by mapping the problem of base flipping to a diffusion of reaction coordinate on an underlying free energy landscape from which we calculate the time scale of looping out of bulge base. We find that the time scale increases upon increasing pressure and changes dramatically at 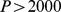 atm. We associate this behavior to increased solvation of the bulge base at high pressures.

The effect of hydration, namely the increased hydration level of the bulge bases led to slowing down of the bulge flipping kinetics. It implies that the structural changes in water and the RNA hydration at high pressures may be relevant to self-catalytic properties of small RNAs. Indeed, water exhibits many anomalous behavior [33], [34] as a function of pressure and temperature, including local structural changes in the liquid state [32]. It would be important to explore the kinetics of bulge bases as a function of pressure and temperature to see if certain range of pressure and temperature may facilitate the kinetics of bulge bases. Moreover, recent experimental studies suggest that in the presence of a temperature gradient, biopolymers such as RNA, DNA can be separated and selected depending on their shape, size and sequence apart from earlier known accumulation and depletion behaviors [35]. The vents create porous precipitates that have large connected pores. Adding to this is the fact that surfaces affect the local structure of water and hence may affect the hydration of RNAs leading to changes in the kinetics [36]. In future, it would be important to explore not only the effect of thermodynamic conditions but also the effect of surfaces on the kinetics.
